# The longitudinal associations between appearance-focused social network site use and body dissatisfaction among college students: serial mediation of appearance comparison and internalization of appearance ideals

**DOI:** 10.3389/fpsyt.2026.1892412

**Published:** 2026-07-01

**Authors:** Tianyu Zhang, Xuerong Zhao, Chong Han, Yinghang Huang, Xiangkui Zhang

**Affiliations:** 1 School of Education, Changchun Normal University, Changchun, China; 2 School of Psychology, Northeast Normal University, Changchun, China; 3 School of Educational Science and Technology, Anshan Normal University, Anshan, China; 4 School of Education Science, Inner Mongolia Minzu University, Tongliao, China

**Keywords:** appearance comparison, body dissatisfaction, internalization of appearance ideals, self-objectification, social network site use

## Abstract

**Introduction:**

Body dissatisfaction is common among college students, and social network site use is a potential risk factor. However, research rarely focuses on specific content types. This study investigates how appearance-focused social network site use affects body dissatisfaction and its underlying mechanisms.

**Methods:**

This longitudinal study included 1,999 college students assessed at three time points over one year. It examined the longitudinal associations of appearance-focused social network site use with body dissatisfaction, as well as the mediating roles of appearance comparison, internalization of appearance ideals, and self-objectification.

**Results:**

Appearance-focused social network site use was significantly and positively prospectively associated with body dissatisfaction in college students. Appearance comparison and internalization of appearance ideals independently mediated this relationship, and their serial mediation effect was significant. The independent mediating effect of self-objectification and chain paths involving this variable were not significant.

**Discussion:**

These findings support the relevance of classic theories in the new media context and offer practical insights for interventions to reduce college students’ body dissatisfaction.

## 1 Introduction

Body dissatisfaction involves negative feelings about one’s own body. It reflects dissatisfaction with specific physical features or overall appearance, involving cognitive, affective, and behavioral experiences (1). College students transition from adolescence to early adulthood. During this period, body image, self-identity, and social identity develop significantly (2). Body dissatisfaction leads to adverse outcomes, such as distorted self-concept, depression, lower well-being, and eating disorders (3, 4). To understand this issue and create interventions, researchers examine risk factors. Social network site use is a prominent factor in this context (5). Social network sites have changed how youth interact socially. Their algorithm-driven content recommendations are now part of students’ daily routines (6). Students often browse appearance-related content or use image-editing tools for self-presentation. This digital body presentation is a common media behavior (7). However, idealized aesthetic standards on social network sites interact with college students’ aesthetic sensitivity. Prolonged exposure to this curated environment induces cognitive biases, leading individuals to focus excessively on and negatively evaluate their own appearance. This process increases the likelihood of appearance comparison and internalization of appearance ideals, which ultimately leads to body dissatisfaction. Therefore, this study investigates how appearance-focused social network site use affects college students’ body dissatisfaction and its underlying mechanisms.

## 2 Literature review

### 2.1 Appearance-focused social network site use and body dissatisfaction

Studies on the relationship between social network site use and body dissatisfaction show mixed results. Some suggest that social network site use leads to body dissatisfaction. According to the tripartite influence model, media, family, and peers influence body dissatisfaction (8). Social network sites combine media attributes with peer interactions. This structure creates frequent opportunities for appearance comparison, prompting individuals to internalize the “thin ideal” and evaluate their own appearance (9). For example, studies found positive links between social network site use and both thin-ideal internalization and disordered eating behaviors among adolescents and college students (10). Conversely, some argue that social network site use may reduce body dissatisfaction. One study found a positive link between usage duration and body satisfaction (11). These mixed findings may result from differences in how researchers measure different types of social network site use (7). The impact of social network site use on body satisfaction depends largely on specific content related to physical appearance. For example, compared to general usage metrics, interacting with appearance-related content leads to frequent appearance comparison. Also, appearance-related feedback triggers negative body image more often than general feedback (12).

However, to our knowledge, research specifically examining appearance-focused social network site use remains limited. Existing studies show limitations in measurement methods. For example, some studies used Instagram and Snapchat usage frequency as indicators (13, 14), while others measured daily TikTok usage duration (15). Although these platforms focus on images, they cover diverse topics beyond physical appearance, such as travel and education. Consequently, general usage frequency alone may not accurately reflect appearance-focused social network site use, highlighting the need for a more precise measurement. Content analysis studies indicate that appearance-focused social network site content primarily emphasizes personal appearance, body shape, and dressing style (16, 17). Such content often uses filters, digital retouching, and specific hashtags to elicit appearance-related attention and interaction. Exposure to this content triggers appearance comparison and internalization of appearance ideals, subsequently leading to body dissatisfaction. Accordingly, this study defines appearance-focused social network site use as the extent of user exposure to content emphasizing personal appearance, body shape, and dressing style. Building upon this, the present study differentiates types of social network site content and examines the link between appearance-focused social network site use and body dissatisfaction among college students. Thus, Hypothesis 1 (H1) states that appearance-focused social network site use significantly and positively predicts college students’ body dissatisfaction.

### 2.2 The mediating role of appearance comparison

Appearance comparison likely mediates the relationship between appearance-focused social network site use and body dissatisfaction. Social comparison theory states that when individuals lack objective standards for self-assessment, they tend to evaluate themselves by comparing with others (18). A higher tendency for appearance comparison increases the risk of body dissatisfaction. Studies found a significant link between appearance comparison and concerns about weight and body shape (19). The visual nature of social network sites increases opportunities for appearance comparison. Also, users often present themselves through photos, which allows for continuous evaluation of appearance. The research found that appearance comparisons on social network sites link more strongly to negative body image than comparisons in traditional media or daily offline settings (20). Longitudinal research also indicated that appearance comparison on Facebook predicted later body dissatisfaction (21). Hypothesis 2 (H2) states that appearance comparison mediates the relationship between appearance-focused social network site use and body dissatisfaction among college students.

### 2.3 The mediating role of internalization of appearance ideals

Internalization of appearance ideals may also mediate the relationship between appearance-focused social network site use and body dissatisfaction. The tripartite influence model defines internalization of appearance ideals as the process where individuals adopt and integrate societal aesthetic standards, body shape expectations, and norms into their own values (8). Many studies explore how internalization of appearance ideals mediates the link between media use and body image. For example, internalization of appearance ideals mediated the link between sexually objectifying media use and body surveillance in female adolescents (22). In another study, general ideal internalization mediated the link between viewing others’ selfies and facial dissatisfaction (23). Recent longitudinal research indicated that photo-based social network site activities affected adolescents’ body image through thin-ideal internalization (14). Hypothesis 3 (H3) states that internalization of appearance ideals mediates the relationship between appearance-focused social network site use and body dissatisfaction among college students.

### 2.4 The mediating role of self-objectification

Self-objectification may also mediate the relationship between appearance-focused social network site use and body dissatisfaction. Objectification theory states that prolonged exposure to objectifying environments leads individuals to view themselves as objects, forming a self-objectification mechanism of continuous self-monitoring (24). Digital platforms often show students content about beauty and slimness. This environment reinforces objectifying experiences and may promote the process of self-objectification (25). Studies found a link between social platform use and self-objectification (26), and exposure to objectifying content immediately raises self-objectification levels (27). Individuals with high self-objectification often monitor their bodies excessively, which leads to negative experiences like anxiety and body shame (28). Hypothesis 4 (H4) states that self-objectification mediates the relationship between appearance-focused social network site use and body dissatisfaction among college students.

### 2.5 Relationships among mediating variables

Appearance comparison, internalization of appearance ideals, and self-objectification may function in specific sequences rather than independently. First, appearance comparison may facilitate internalization of appearance ideals. For instance, one study found that social network site use predicted body comparison and internalization of appearance ideals, which in turn predicted appearance self-esteem, even after controlling for media, family, and peer pressures (29). Second, internalization of appearance ideals may lead to self-objectification. Previous research showed that body talk on Facebook influenced adolescents’ objectifying behaviors through the internalization of reward beauty and self-objectification (30). Another study found that online objectification experiences predicted body shame in female college students through the serial mediating effects of internalization of ideal beauty and self-objectification (31). Third, appearance comparison may trigger self-objectification. According to the tripartite influence model and objectification theory, individuals who engage in appearance comparison evaluate both the comparison target’s body and their own body from an observer’s perspective, which triggers self-objectification. Finally, a developmental sociocultural framework combines social comparison theory, objectification Theory, and social network site-related variables to explain how social network site use impacts body dissatisfaction (32). However, empirical evidence for this model remains lacking. Based on the aforementioned theoretical and empirical studies, Hypothesis 5 (H5) states that appearance comparison, internalization of appearance ideals, and self-objectification serially mediate the relationship between appearance-focused social network site use and body dissatisfaction.

**Figure 1:**
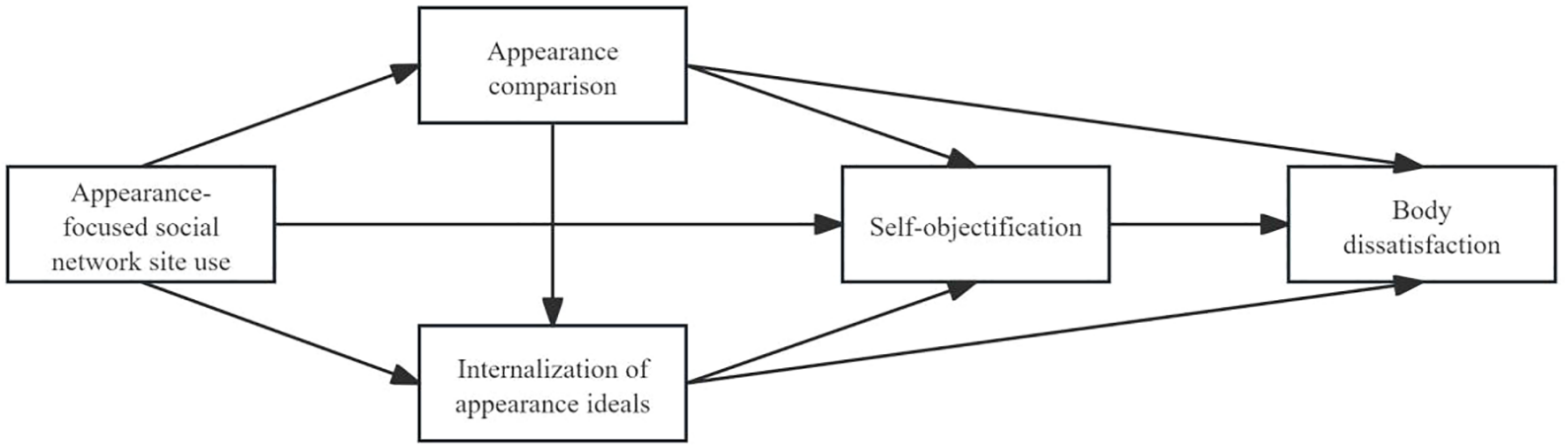
Hypothetical model diagram.

Although research has examined the relationship between social network site use and body dissatisfaction, several limitations remain. First, previous studies primarily focused on general social network site use (e.g., duration or frequency) without differentiating content types, which may obscure the predictive utility of appearance-focused social network site use. Second, previous mechanism studies often examined isolated mediating variables rather than testing the sequential pathway of “appearance comparison → internalization of appearance ideals → self-objectification.” Finally, previous studies used cross-sectional designs, preventing the determination of temporal relationships. To address these limitations, the present study employed a longitudinal design to investigate the longitudinal association between appearance-focused social network site use and body dissatisfaction among college students. The study examined the independent and chain mediating roles of appearance comparison, internalization of appearance ideals, and self-objectification, and a model diagram of the research hypotheses is put forward in **Figure 1**. This approach clarifies how social network site use influences body dissatisfaction and extends existing theoretical frameworks.

## 3 Method

### 3.1 Participants

This study used convenience sampling to recruit college students from five universities in Changchun, Daqing, and Anshan. Inclusion criteria were (1): current enrollment as a college student, and (2) voluntary participation with signed informed consent. The study used a one-year longitudinal design with three measurement time points (T1, T2, and T3) and a 6-month interval between assessments. Baseline data collection (T1) occurred in October 2024. Initially, 2,745 participants meeting the inclusion criteria were recruited. After obtaining institutional ethical approval and participant consent, the study collected data via an online survey platform. Participants completed each assessment in approximately 3 to 5 minutes. All items were mandatory to ensure completeness, resulting in no missing data.

The study applied strict data cleaning criteria to ensure data quality: (1) excluding 73 participants who failed an embedded attention check item (e.g., “Please select ‘agree’ for this question”); (2) excluding 40 participants with response times under 30 seconds; (3) excluding 57 duplicate submissions identified by student ID and IP address; and (4) excluding 111 participants who selected the same option for six consecutive items, indicating patterned responding. After data cleaning, the T1 baseline sample included 2,464 participants. At T2, 348 participants dropped out due to school dropout, transfer, illness, or failing data cleaning criteria, leaving 2,116 participants. At T3, an additional 117 participants dropped out for the same reasons, yielding a final sample of 1,999 participants. To test for systematic attrition, the study compared key variables before dropout between dropouts and remaining participants. Specifically, the analysis compared T1 variables between T2 dropouts and remaining participants, and T1 and T2 variables between T3 dropouts and remaining participants. Results showed no significant differences (*ts* < 1.96, *ps* > 0.05), indicating random attrition. The final valid sample comprised 1,999 participants (875 males, 43.8%; 1,124 females, 56.2%), with a mean baseline age of 18.39 ± 0.68 years and a mean BMI of 20.78 ± 3.29.

### 3.2 Measures

#### 3.2.1 Appearance-focused social network site use

The current study used the Short Interactions with Positive Social Media Content Scales (33, 34). The questionnaire was adapted into Chinese using the translation-back-translation method (35). First, a doctoral student in psychology translated the original scale. Second, an English professor back-translated the draft. Third, another doctoral student in psychology compared the versions and corrected the terminology. Fourth, four doctoral students evaluated and revised the items for clarity and professionalism. Finally, a senior psychology professor and the research team finalized the translation to ensure accuracy and cultural appropriateness.

This measure assessed how frequently participants browsed content related to “beautiful or handsome appearance” “slim or muscular body” and “nice clothing style” on social network sites to evaluate their exposure to appearance-related content. The measure uses a 5-point Likert scale ranging from 1 (never) to 5 (always), with higher scores indicating a higher frequency of browsing appearance-focused social network site content. In the current study, the Cronbach’s α coefficient for all items at T1 was 0.83.

#### 3.2.2 Appearance comparison

The Social Network Site Appearance Comparison Scale was used to measure participants’ tendency to engage in appearance comparison with others while using social network sites (11). Participants rated their agreement with three items: “When using social network sites, I compare my physical appearance to others,” “When using social network sites, I compare how I dress to others,” and “When using social network sites, I compare my body shape to others.” Responses were recorded on a 5-point Likert scale ranging from 1 (strongly disagree) to 5 (strongly agree). The questionnaire demonstrated good reliability, with a Cronbach’s α coefficient of 0.87 for all items at T2.

#### 3.2.3 Internalization of appearance ideals

The General Attractiveness Internalization subscale of the Sociocultural Attitudes Towards Appearance Questionnaire was used to measure participants’ internalization of appearance ideals (36). The subscale consisted of six items, such as “It is important for me to look attractive.” Responses were rated on a 5-point Likert scale ranging from 1 (strongly disagree) to 5 (strongly agree), with higher scores indicating a higher level of internalization of appearance ideals. The Cronbach’s α coefficient for all items at T2 was 0.86.

#### 3.2.4 Self-objectification

The Body Surveillance subscale of the Objectified Body Consciousness Scale, developed by McKinley and Hyde (37) and revised by Jackson and Chen (38), was used to measure participants’ self-objectification. The scale comprised eight items, such as “I often think about how I look several times a day.” Responses were recorded on a 7-point scale, where higher scores indicated higher levels of self-objectification. The Cronbach’s α coefficient for all items at T2 was 0.85.

#### 3.2.5 Body dissatisfaction

The Body Areas Satisfaction Scale was used to measure participants’ body dissatisfaction (39). The scale consisted of nine items that assessed satisfaction with different body areas (e.g., waist, thighs, and abdomen) and overall appearance. Responses were recorded on a 5-point Likert scale ranging from 1 (very dissatisfied) to 5 (very satisfied). Item scores were reverse-coded and then averaged to yield a total score, with higher scores representing greater body dissatisfaction. The questionnaire demonstrated good reliability, with a Cronbach’s α coefficient of 0.92 for all items at T3.

#### 3.2.6 Control variables

The Social Network Site Use Intensity Scale developed by Ellison et al. was used (40). This scale comprised eight items, such as “Social network sites are a part of my everyday activity.” The Cronbach’s α coefficient for all items was 0.83. Additionally, gender and Body Mass Index (BMI) were included as control variables.

### 3.3 Data analysis

This study used SPSS 26.0 for all data analyses. First, the study computed descriptive statistics for the primary variables and examined correlations among control and core variables. Next, Hayes’ PROCESS macro (Model 6) tested the independent and serial mediating effects of appearance comparison, internalization of appearance ideals, and self-objectification on the relationship between appearance-focused social network site use and body dissatisfaction. These analyses controlled for gender, BMI, and general social network site use intensity. The percentile bootstrap method estimated the 95% confidence intervals for the indirect effects.

## 4 Results

### 4.1 Common method bias analysis

Harman’s single-factor test assessed the presence of common method bias. The analysis revealed nine factors with eigenvalues greater than 1; the first factor accounted for 15.34% of the total variance. As this value is below the 40% threshold, common method bias was not a concern in the current data.

### 4.2 Descriptive statistics and correlation analysis

The study calculated descriptive statistics and correlations among gender, body mass index (BMI), social network site use intensity, and the primary study variables (see **Table 1**). The results indicated that gender was significantly and positively correlated with T1 appearance-focused social network site use, T2 internalization of appearance ideals, and T2 self-objectification. BMI was significantly and positively correlated with body dissatisfaction. Furthermore, social network site use intensity was significantly and positively correlated with all primary study variables. Consequently, subsequent analyses controlled for gender, BMI, and social network site use intensity. Additionally, T1 appearance-focused social network site use was significantly and positively correlated with T2 appearance comparison, T2 internalization of appearance ideals, T2 self-objectification, and T3 body dissatisfaction. Finally, T2 appearance comparison, T2 internalization of appearance ideals, and T2 self-objectification were all significantly and positively correlated with T3 body dissatisfaction.

**Table 1:** Descriptive statistics and correlation analysis.

Variable	*M (SD)*	1	2	3	4	5	6	7	8
1 Gender	0.44 (0.50)	1							
2 BMI	20.78 (3.29)	-0.092^**^	1						
3 SNS	3.14 (0.69)	0.107^**^	0.021	1					
4 T1 AF-SNS	3.01 (0.82)	0.158^**^	-0.021	0.347^**^	1				
5 T2 AC	2.16 (0.75)	0.013	0.016	0.242^**^	0.310^**^	1			
6 T2 AII	3.50 (0.49)	0.097^**^	-0.029	0.212^**^	0.194^**^	0.199^**^	1		
7 T2 SO	3.90 (0.75)	0.050^*^	-0.006	0.195^**^	0.184^**^	0.224^**^	0.243^**^	1	
8 T3 BD	2.89 (0.43)	0.028	0.053^*^	0.090^**^	0.123^**^	0.114^**^	0.103^**^	0.079^**^	1

N = 1999; the Gender is a dummy variable (Male = 1, Female = 0); ^*^p < 0.05, ^**^p < 0.01; BMI, Body Mass Index; SNS, Social networking site intensity; T1 AF-SNS, Time 1 Appearance-focused social network site use; T2 AC, Time 2 Appearance Comparison; T2 AII, Time 2 Internalization of appearance ideals; T2 SO, Time 2 Self-objectification; T3 BD, Time 3 Body dissatisfaction. The same applies below.

### 4.3 Testing for mediation

Building upon the correlation analysis and following previous research, this study conducted regression analyses to examine the effect of appearance-focused social network site use on body dissatisfaction, controlling for gender, body mass index (BMI), and social network site use intensity (see **Table 2**).

**Table 2:** Regression analyses for the mediation model.

Outcome	Predictor	*B*	*SE*	*95% CI* for *B*	*t*	*p*
T3 BD						
Model summary	*R* = 0.144, *R²* = 0.021, Adj. *R²* = 0.019, *F*_(4, 1994)_ = 10.49, *p* < 0.001					
	Gender	0.029	0.060	[-0.090, 0.148]	0.48	0.632
	BMI	0.055	0.022	[0.011, 0.098]	2.46	0.014
	SNS	0.052	0.024	[0.005, 0.098]	2.18	0.029
	T1 AF-SNS	0.104	0.024	[0.057, 0.151]	4.37	< 0.001
T2 AC						
Model summary	*R* = 0.344, *R²* = 0.119, Adj. *R²* = 0.117, *F*_(4, 1994)_ = 67.12, *p* < 0.001					
	Gender	-0.116	0.057	[-0.229, -0.004]	-2.03	0.043
	BMI	0.014	0.021	[-0.027, 0.056]	0.69	0.493
	SNS	0.155	0.022	[0.111, 0.199]	6.91	< 0.001
	T1 AF-SNS	0.263	0.023	[0.219, 0.307]	11.62	< 0.001
T2 AII						
Model summary	*R* = 0.286, *R²* = 0.082, Adj. *R²* = 0.080, *F*_(5, 1993)_ = 35.44, *p* < 0.001					
	Gender	0.169	0.059	[0.054, 0.284]	2.88	0.004
	BMI	-0.026	0.022	[-0.069, 0.016]	-1.22	0.221
	SNS	0.141	0.023	[0.096, 0.187]	6.09	< 0.001
	T1 AF-SNS	0.092	0.024	[0.045, 0.139]	3.86	< 0.001
	T2 AC	0.135	0.023	[0.091, 0.180]	5.93	< 0.001
T2 SO						
Model summary	*R* = 0.328, *R²* = 0.108, Adj. *R²* = 0.105, *F*_(6, 1992)_ = 40.12, *p* < 0.001					
	Gender	0.024	0.058	[-0.090, 0.138]	0.41	0.679
	BMI	-0.003	0.021	[-0.045, 0.039]	-0.13	0.894
	SNS	0.097	0.023	[0.052, 0.143]	4.21	< 0.001
	T1 AF-SNS	0.069	0.024	[0.023, 0.116]	2.94	0.003
	T2 AC	0.143	0.023	[0.099, 0.188]	6.29	< 0.001
	T2 AII	0.179	0.022	[0.136, 0.223]	8.13	< 0.001
T3 BD						
Model summary	*R* = 0.176, *R²* = 0.031, Adj. *R²* = 0.028, *F*_(7, 1991)_ = 9.10, *p* < 0.001					
	Gender	0.026	0.060	[-0.093, 0.144]	0.43	0.670
	BMI	0.055	0.022	[0.012, 0.099]	2.50	0.012
	SNS	0.027	0.024	[-0.020, 0.075]	1.12	0.262
	T1 AF-SNS	0.075	0.025	[0.027, 0.124]	3.06	0.002
	T2 AC	0.064	0.024	[0.017, 0.111]	2.69	0.007
	T2 AII	0.063	0.023	[0.017, 0.109]	2.69	0.007
	T2 SO	0.030	0.023	[-0.016, 0.075]	1.27	0.205

N = 1,999. B represents the unstandardized regression coefficient. CI refers to the 95% confidence interval for B.

**Figure 2:**
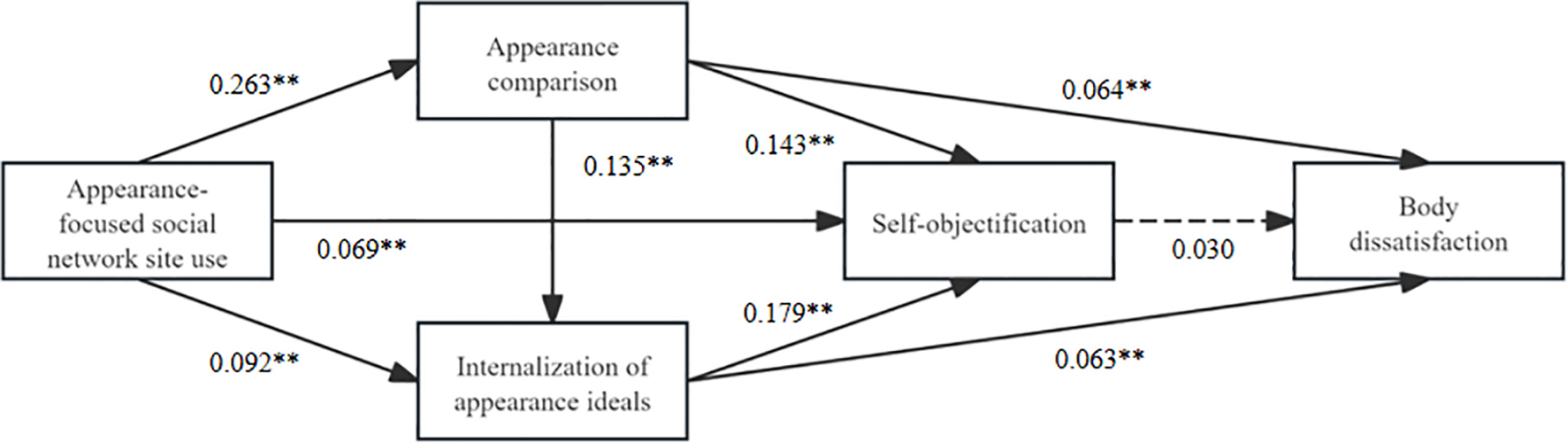
Mediating effect model of appearance-focused social network site use on body dissatisfaction. **p < 0.01.

As shown in **Table 2**, the total-effect model indicated that T1 appearance-focused social network site use significantly and positively predicted T3 body dissatisfaction after controlling for gender, BMI, and social network site intensity. In the mediation model, T1 appearance-focused social network site use significantly predicted T2 appearance comparison, T2 internalization of appearance ideals, and T2 self-objectification. T2 appearance comparison significantly predicted T2 internalization of appearance ideals, T2 self-objectification, and T3 body dissatisfaction. T2 internalization of appearance ideals significantly predicted T2 self-objectification and T3 body dissatisfaction. T2 self-objectification did not significantly predict T3 body dissatisfaction. The path coefficients among the variables and the model diagram are illustrated in **Figure 2**.

Subsequently, the percentile bootstrap method was employed to test the significance of indirect effects, with an effect considered significant if the 95% confidence interval (CI) did not contain zero. Regarding the independent mediating pathways, the indirect effects of T1 appearance-focused social network site use on T3 body dissatisfaction were significant through T2 appearance comparison (effect size = 0.017, SE = 0.007, 95%CI[0.004, 0.031]) and T2 internalization of appearance ideals (effect size = 0.006, SE = 0.003, 95%CI[0.001, 0.012]). Conversely, the indirect effect through T2 self-objectification was not significant (95%CI[-0.002, 0.007]). For the serial mediating pathways, only the sequential indirect effect of T1 appearance-focused social network site use on T3 body dissatisfaction via the path “T2 appearance comparison → T2 internalization of appearance ideals” was significant (effect size = 0.002, SE = 0.001, 95%CI[0.001, 0.004]). The remaining serial pathways were not significant: the path via T2 appearance comparison to T2 self-objectification (95% CI [-0.001, 0.003]), the path via T2 internalization of appearance ideals to T2 self-objectification (95% CI [0.000, 0.002]), and the three-mediator serial pathway (95% CI [0.000, 0.001]). **Table 3** details the effect decomposition and relative effect sizes for the significant pathways. The full set of theoretically tested indirect pathways, including non-significant pathways, is reported in **Supplementary Table 1**.

**Table 3:** Analysis of mediating effects.

Effect	Effect size	Boot SE	Boot 95% CI	Relative effect value
Direct	0.075	0.025	0.027-0.124	72.12%
Indirect	0.029	0.008	0.014-0.044	27.88%
Indirect effect 1	0.017	0.007	0.004-0.031	16.35%
Indirect effect 2	0.006	0.003	0.001-0.012	5.77%
Indirect effect 3	0.002	0.001	0.001-0.004	1.92%

Boot SE and Boot 95% CI refer to the standard error and 95% confidence interval of the indirect effects estimated by the percentile bootstrap method, respectively. Indirect effect 1: appearance-focused social network site use → appearance comparison → body dissatisfaction; Indirect effect 2: appearance-focused social network site use → internalization of appearance ideals → body dissatisfaction; Indirect effect 3: appearance-focused social network site use → appearance comparison → internalization of appearance ideals → body dissatisfaction.

## 5 Discussion

Guided by the tripartite influence model and objectification theory, this longitudinal study examined how appearance-focused social network site use affects body dissatisfaction among college students and the underlying mechanisms. The results indicated support for the theoretical hypotheses and extended previous research, clarifying how body image concerns develop in digital media contexts.

### 5.1 The direct prospective association of appearance-focused social network site use with body dissatisfaction

After controlling for gender, BMI, and general social network site use intensity, T1 appearance-focused social network site use was significantly and positively prospectively associated with T3 body dissatisfaction, supporting Hypothesis 1. This finding aligns with previous research (7). According to sociocultural theory, social media platforms use algorithmic recommendation systems to create a visual environment filled with idealized images. Repeated exposure to this curated and beautified appearance-related content, such as the “pale, young, and thin” aesthetic on Chinese platforms like Xiaohongshu and Douyin, directly drives perceptual and cognitive processing.This process increases attentional bias toward appearance cues and strengthens the weight of the “ideal appearance” in self-evaluation. Meanwhile, continuous exposure gradually narrows individuals’ perceptual boundaries regarding what constitutes a “normal” or “acceptable” appearance. Consequently, this raises self-scrutiny standards and increases the risk of dissatisfaction (41).

The findings further clarify the difference in predictive power between appearance-focused social network site use and general media exposure. General social interactions lack direct visual triggers for body image, making it difficult to activate appearance-related self-scrutiny. In contrast, appearance-focused social network site use specifically captures engagement with visual stimuli (14). The data support this notion. Although social network site use intensity correlated with body dissatisfaction, it did not directly predict T3 body dissatisfaction after controlling for appearance-focused social network site use. This indicates that exposure to appearance-related content, rather than general metrics like usage duration or frequency, determines the risk of body dissatisfaction.

### 5.2 The independent and chain mediating roles of appearance comparison and internalization of appearance ideals

Consistent with Hypotheses 2 and 3, appearance comparison and internalization of appearance ideals independently mediated the relationship between appearance-focused social network site use and college students’ body dissatisfaction, and their chain mediating effect was significant. This finding further supports the application of the tripartite influence model within the digital media context.

First, social networking sites create a space for upward social comparison. Users present idealized images, prompting individuals to evaluate themselves through social comparison. Frequent exposure to these idealized images leads users to compare themselves against these standards. Such persistent upward comparison subsequently is associated with body dissatisfaction (42). Second, social network sites continuously present idealized attractiveness standards through algorithmic recommendations. Repeated exposure to this content caused individuals to gradually internalize these aesthetic norms (43). This internalization process is particularly prominent within the Chinese collectivist cultural context. Due to strong “face consciousness”, conforming to peer-approved appearance standards is often crucial for gaining social acceptance. When individuals perceive a discrepancy between their own appearance and these internalized standards, it triggers self-ideal discrepancies, which in turn exacerbate body dissatisfaction.

More importantly, the significant serial mediating pathway shows that appearance-focused social network site use influence body dissatisfaction through a sequential socio-cognitive mechanism. This mechanism reflects a shift from visual stimuli to cognitive construction. When exposed to appearance content, individuals first engage in appearance comparison. This comparison reinforces the perceived authority of aesthetic standards, which provides a basis for the internalization of appearance ideals. Consequently, external pressure transforms into body dissatisfaction (11). This finding provides empirical support for a developmental sociocultural framework (32) by clarifying the central roles of appearance comparison and internalization of appearance ideals.

### 5.3 The non-significant mediating role of self-objectification

Self-objectification did not independently mediate the relationship between appearance-focused social network site use and body dissatisfaction. Chain mediating pathways involving self-objectification were also non-significant; therefore, Hypothesis 4 and 5 lacked support. Unlike short-term experiments that show immediate effects of self-objectification on state body dissatisfaction, this study examined trait-level changes over one year. In this longitudinal framework, appearance comparison and internalization of appearance ideals are likely more stable and fundamental cognitive risk factors. Although self-objectification correlates with these variables, it primarily manifests as body surveillance (38). This tendency induces immediate emotional fluctuations. Without the deep internalization of aesthetic standards, transient emotional responses do not solidify into long-term body dissatisfaction. Consequently, self-objectification may act as a concomitant tendency rather than a core mediating mechanism for trait body dissatisfaction (44).In other words, appearance comparison and internalization of appearance ideals may have overshadowed the predictive effect of self-objectification on body dissatisfaction.

An alternative explanation concerns the operational definition of self-objectification. This study measured self-objectification using the body surveillance subscale of the objectified body consciousness scale. This subscale primarily assesses how frequently individuals monitor their appearance to conform to external standards. According to objectification theory, body surveillance is a proximal predictor of body shame. However, the dependent variable in this study was body dissatisfaction, which reflects a relatively stable negative cognitive evaluation of one’s body shape or appearance features. Although body shame and body dissatisfaction are conceptually highly related, body shame primarily involves immediate emotional reactions, whereas body dissatisfaction involves enduring cognitive biases (45). This difference in cognitive depth may explain why self-objectification did not significantly predict body dissatisfaction. Future research should use multidimensional measures to further examine this pathway.

### 5.4 Theoretical and practical implications

These findings offer theoretical and practical implications. Theoretically, this study sheds light on how social network site use affects body image and provides longitudinal support for the tripartite influence model in a cross-cultural context. Specifically, cognitive evaluation processes (appearance comparison and internalization of appearance ideals) drive body dissatisfaction more fundamentally than self-monitoring (self-objectification). Practically, these findings can guide the development of body image interventions for college students. Mental health educators can use this model to design cognitive-behavioral interventions that reduce appearance comparison and internalization of appearance ideals, alleviating body dissatisfaction. Furthermore, social networking platforms should optimize algorithms and establish content-rating and filtering systems. Reducing overexposure to idealized appearance content at the source can create a more inclusive digital environment. Finally, college students should improve their media literacy to manage their exposure to social network site content. By recognizing and disrupting appearance comparison behaviors during daily use, individuals may break the pathway from appearance comparison to internalization of appearance ideals.

### 5.5 Limitations and future directions

Despite these contributions, this study has several limitations. First, although this study used a three-wave longitudinal design, it did not measure all core variables at each wave. Specifically, the three mediating variables were measured only at T2, limiting rigorous empirical support for their temporal sequence. Furthermore, because the model did not control for T1 body dissatisfaction or baseline levels of the mediating variables, the current findings are more appropriately described as prospective associations rather than evidence of causal effects. Future research should measure all variables at each time point and use cross-lagged panel models to clarify temporal trajectories and dynamic influences. Second, the theoretical model tested unidirectional relationships and could not assess potential bidirectional cycles between appearance-focused social network site use and body dissatisfaction. Research shows that body image disturbances may reinforce dependence on appearance-related media content (46). Future studies could use experience sampling to track daily changes and analyze the dynamic feedback between media exposure and body image. Third, this study primarily measured the passive browsing dimension of social network site use and did not capture active interactions. Because interactive behaviors (e.g., liking and commenting) may affect body image through social reinforcement, future research should combine objective platform usage logs with experimental methods. This approach can distinguish how different usage modes affect body dissatisfaction. Fourth, this study only used the body surveillance subscale to measure self-objectification, which may not capture all dimensions of this construct. Future research should use multidimensional measures to clarify how self-objectification affects body dissatisfaction over time. Lastly, this study used body satisfaction measures developed in Western contexts, which may not fully reflect cultural differences in body image. Eastern culture traditionally emphasizes facial features and a slender physique, whereas Western culture emphasizes muscularity (47). Future research should use culturally sensitive measures and conduct cross-cultural comparisons.

## 6 Conclusion

Using a one-year longitudinal design, this study examined how appearance-focused social network site use affects college students’ body dissatisfaction and the underlying mechanisms. The main conclusions are: (1) Appearance-focused social network site use was significantly and positively prospectively associated with body dissatisfaction among college students. (2) Appearance-focused social network site use showed longitudinal associations with body dissatisfaction through the independent mediating effects of appearance comparison and internalization of appearance ideals, and their chain mediating effect. (3) The independent mediating effect of self-objectification and the chain mediating effects involving this variable were non-significant. These findings suggest that within a longitudinal framework, appearance comparison and internalization of appearance ideals are more significant cognitive risk factors than self-objectification.

## Data Availability

The raw data supporting the conclusions of this article will be made available by the authors, without undue reservation.
