# Supplementary material for: The longitudinal associations between appearance-focused social network site use and body dissatisfaction among college students: serial mediation of appearance comparison and internalization of appearance ideals
Source: Front Psychiatry. 2026 Jul 1;17:1892412. doi: 10.3389/fpsyt.2026.1892412 (PMC13368731; doi:10.3389/fpsyt.2026.1892412)
Supplement: Supplementary file 1 [file Table1.docx]

**Supplementary Information**

**Table S1** Full results of all theoretically tested indirect effects

| **Indirect effect** | **Pathway** | **Effect** | **Boot SE** | **Boot 95% CI** |
| --- | --- | --- | --- | --- |
| Ind1 | T1 AF-SNS → T2 AC → T3 BD | 0.017 | 0.007 | [0.004, 0.031] |
| Ind2 | T1 AF-SNS → T2 AII → T3 BD | 0.006 | 0.003 | [0.001, 0.012] |
| Ind3 | T1 AF-SNS → T2 SO → T3 BD | 0.002 | 0.002 | [-0.002, 0.007] |
| Ind4 | T1 AF-SNS → T2 AC → T2 AII → T3 BD | 0.002 | 0.001 | [0.001, 0.004] |
| Ind5 | T1 AF-SNS → T2 AC → T2 SO → T3 BD | 0.001 | 0.001 | [-0.001, 0.003] |
| Ind6 | T1 AF-SNS → T2 AII → T2 SO → T3 BD | 0.000 | 0.000 | [0.000, 0.002] |
| Ind7 | T1 AF-SNS → T2 AC → T2 AII → T2 SO → T3 BD | 0.000 | 0.000 | [0.000, 0.001] |

Note. T1 AF-SNS = Time 1 appearance-focused social network site use; T2 AC = Time 2 appearance comparison; T2 AII = Time 2 internalization of appearance ideals; T2 SO = Time 2 self-objectification; T3 BD = Time 3 body dissatisfaction. Boot SE and Boot 95% CI refer to the standard error and 95% confidence interval estimated by the percentile bootstrap method based on 5,000 bootstrap samples. An indirect effect was interpreted as significant when the 95% CI did not include zero.
